# Systematic literature review and assessment of patient-reported outcome instruments in sickle cell disease

**DOI:** 10.1186/s12955-018-0930-y

**Published:** 2018-05-21

**Authors:** Grammati Sarri, Menaka Bhor, Seye Abogunrin, Caroline Farmer, Savita Nandal, Rashid Halloway, Dennis A. Revicki

**Affiliations:** 1 Evidera, Metro Building, 6th Floor, 1 Butterwick, London, W6 8DL UK; 2 Novartis, One Health Plaza, East Hanover, NJ 07936-1080 USA; 3 Evidera, 7101 Wisconsin Ave, Suite 1400, Bethesda, MD 20814 USA

**Keywords:** Sickle cell disease, Patient-reported outcomes, Psychometric properties, COSMIN

## Abstract

**Background:**

Sickle cell disease (SCD) is a chronic condition associated with high mortality and morbidity. It is characterized by acute clinical symptoms such as painful vaso-occlusive crises, which can impair health-related quality of life (HRQL). This study was conducted to identify validated patient-reported outcome (PRO) instruments for use in future trials of potential treatments for SCD.

**Methods:**

A systematic literature review (SLR) was performed using MEDLINE and EMBASE to identify United States (US)-based studies published in English between 1997 and 2017 that reported on validated PRO instruments used in randomized controlled trials and real-world settings. The COnsensus-based Standards for the selection of health Measurement INstruments (COSMIN) checklist was used to assess the quality of PRO instruments.

**Results:**

The SLR included 21 studies assessing the psychometric properties of 24 PRO instruments. Fifteen of those instruments were developed and validated for adults and 10 for children (one instrument was used in both children and young adults aged up to 21 years). Only five of the 15 adult instruments and three of the 10 pediatric instruments were developed specifically for SCD. For most instruments, there were few or no data on validation conducted in SCD development cohorts. Of the 24 PRO instruments identified, 16 had strong internal reliability (Cronbach’s α ≥0.80). There was often insufficient information to assess the content validity, construct validity, responsiveness, or test-retest reliability of the instruments identified for both child and adult populations. No validated PRO instruments measuring caregiver burden in SCD were identified.

**Conclusions:**

The evidence on the psychometric properties of PRO instruments was limited. However, the results of this SLR provide key information on such tools to help inform the design of future clinical trials for patients with SCD in the US.

**Electronic supplementary material:**

The online version of this article (10.1186/s12955-018-0930-y) contains supplementary material, which is available to authorized users.

## Background

Sickle cell disease (SCD) is a lifelong, multisystem condition characterized by hemoglobin polymerization that leads to erythrocyte rigidity, hemolysis, and vaso-occlusion. Prevalence estimates for the United States (US) in 2016 suggest that between 70,000 and 100,000 people had SCD [1, 2]. Also, a further estimated 3.5 million individuals had the sickle cell trait [1, 2], meaning they were carriers of one of several autosomal recessive alleles responsible for the disease. The most common SCD genotype is HbSS, and the disease is most prevalent in people of African ancestry [1].

Vaso-occlusive crises (VOC) and pain associated with such crises are hallmark symptoms in SCD, and typically first manifest in infants around the age of 5 months. These painful episodes can occur without warning and have been described as sharp, intense stabbing or throbbing. The pain can be debilitating, resulting in frequent emergency department (ED) and hospital visits. Furthermore, complications of SCD, such as anemia, infection, stroke can have major physiological, cognitive, and emotional effects on patients [2, 3].

Current US guidelines for SCD management focus mainly on health maintenance and treatment of acute and chronic complications [4]. Health maintenance recommendations include prophylactic penicillin treatment and pneumococcal vaccination in patients with asplenia [4], and screening or diagnostic tests for SCD-related complications; and supportive management includes treatment with antibiotics, pain crisis management, and blood transfusions. Stem cell transplantation is the intervention most likely to be curative, but has many risks and is not performed frequently [5–7]. Because there is currently no pharmacotherapeutic cure for SCD, and in most cases management of the disease is palliative, a key therapeutic goal is to reduce the occurrence of painful crises. For this, the traditional mainstay treatment has been the antineoplastic agent hydroxyurea. This drug helps prevent crises in both adults and children by increasing the amount of fetal hemoglobin found in patients’ red blood cells (RBCs), thus leading to various beneficial effects on RBC structure, content, and function [8, 9]. In turn, this reduces the need for transfusion and the likelihood of organ damage. More recently, an alternative therapy, L-glutamine was also approved by the US Food and Drug Administration (FDA) for the treatment of SCD in children and adults, with the aim of reducing severe SCD-related complications [10].

Despite the availability and use of hydroxyurea and L-glutamine, SCD remains a disease with major unmet needs, with many patients experiencing poor clinical outcomes in both the short and longer term. There is also substantial evidence suggesting that SCD is associated with a considerable impairment of patients’ burden with SCD. However, characterizing the nature and extent of this humanistic deficit, and whether or how it differs between patient subgroups or with disease stage, is hampered by a lack of clarity about which (if any) of the patient-reported outcome (PRO) instruments used to-date are best able to capture patients’ experience of SCD. This lack of clarity has major implications for the investigation into potential new treatments for SCD. In particular, it raises questions about how best to assess whether, or to what extent, such interventions affect humanistic outcomes. Therefore, to inform recommendations of PROs that might be suitable for use in future SCD clinical trials, a systematic literature review (SLR) was conducted to identify, summarize, and evaluate PRO instruments that have been developed and/or validated in previous US trials and observational studies of SCD.

## Methods

### Identification of studies

The SLR was conducted using transparent and reproducible methods, in accordance with standards recommended by the Preferred Reporting Items for Systematic Reviews and Meta-Analyses (PRISMA) Statement [11] and the Cochrane Handbook for Systematic Reviews [12]. A single systematic search was conducted in Embase, Embase In-Process, MEDLINE, and MEDLINE In-Process, to identify studies of interest on PRO instruments, published in English between 1997 and 2017. Specifically, search terms for SCD were combined with terms for psychometric properties of PRO instruments. The search strategy (detailed in Additional file 1) included a combination of free-text search terms and controlled vocabulary terms as recommended by the Cochrane Collaboration [13]. No grey literature conference abstracts were considered for the search because these would have provided inadequate detail for the purposes of the review. Bibliographies of all relevant systematic reviews and/or meta-analyses identified during the search were also reviewed to identify any additional relevant publications.

### Study selection

To identify the most relevant studies for inclusion in the review, publications identified from the electronic database searches were screened against predefined inclusion and exclusion criteria (detailed in Additional file 2) in a two-stage selection process. First, the titles and abstracts of all unique citations from the searches were reviewed against the selection criteria. Second, the full-text versions of all the publications that had been considered relevant at the first stage were assessed to determine which studies should be included in the review. All records were reviewed by one researcher, with validation of 50% of records at both screening levels being performed by a second researcher. A third researcher resolved any discrepancies and confirmed inclusion or exclusion where appropriate.

### Data extraction

Relevant data from the included publications were entered into a standardized predesigned extraction template by one investigator, and then validated by a senior researcher. A third reviewer was consulted to resolve any disagreements.

### Quality assessment

Quality assessment was conducted for the identified PRO instruments by one researcher and validated by a second researcher, using an abbreviated version of the COnsensus-based Standards for the selection of health Measurement INstruments (COSMIN) checklist [14]. This checklist assesses the methodological quality and performance of PRO instruments across various characteristics as reported in psychometric-evaluation studies. The checklist was abbreviated for this study to focus on characteristics that met the FDA criteria [15] for the evaluation of PRO instruments for use in clinical trials: reliability, validity, and responsiveness:

- Reliability is the degree to which the measurement is free from measurement error, as indicated by the extent to which scores for patients who have not changed are the same for repeated measurement under several conditions [14]. Studies were considered to have strong internal consistency reliability when Cronbach’s alpha was ≥0.80;
- Validity is the degree to which a health-related PRO instrument measures the construct it intends to measure [14]. The FDA also considers whether similar patients to those participating in the clinical trial have confirmed the relevance of items in the PRO instrument [16];
- Responsiveness is the ability of a health-related PRO instrument to detect change over time in the construct to be measured [14]. The FDA considers whether responsiveness has been demonstrated in a comparative trial setting [16].

## Results

### Study inclusion

**Fig. 1:**
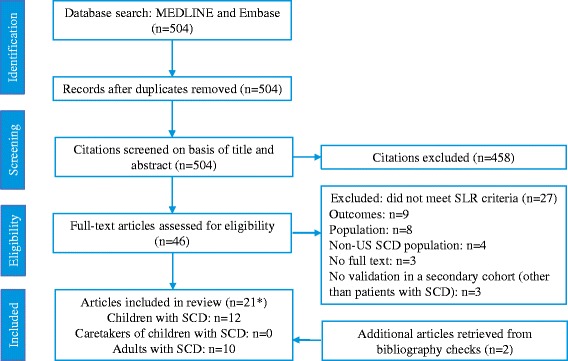
PRISMA Flow Diagram. Abbreviations: PRO = patient-reported outcome; SCD = sickle cell disease; SLR = systematic literature review; US = United States. * Note that one study included an instrument for both children and adults with SCD

The electronic database search yielded 504 unique records. After title and abstract screening, 46 citations were considered relevant for full-text review. Following full-text assessment, 19 studies reporting on the psychometric properties of PRO instruments were identified, and two more articles were added from manual searches of bibliographies of published SLRs. Thus, a total of 21 publications met all inclusion criteria. The selection of studies from the initial search yield to the final number of included studies, using the PRISMA guidelines, is presented in Fig. 1.

### PRO instruments

The 21 studies included in the SLR reported on a total of 24 PRO instruments that had been developed and/or validated in populations with SCD in the US. Fifteen of the instruments (represented in nine publications) were for use with adults, and 10 instruments (in 12 publications) were for children through age 17 years (one instrument was used in both children and young adults aged up to 21 years, and so was included in both populations). No validated PRO instruments designed for caregivers of children with SCD were identified. For most instruments, there were few or no validation data from studies conducted in SCD development cohorts. All studies evaluating adult PRO instruments were cross-sectional studies. Nine studies evaluating pediatric PRO instruments were cross-sectional, and one each was longitudinal, retrospective, or a medical chart review. The most common outcomes evaluated by instruments were coping, self-esteem, or self-efficacy (*n* = 8), followed by health-related quality of life (HRQL; *n* = 5), pain (*n* = 4), and family impact (*n* = 2). Depression, functioning, spirituality, stigma, and treatment satisfaction were each evaluated with one instrument.

### Quality assessment results

As previously mentioned, quality assessment of the PRO instruments was conducted using the abbreviated COSMIN checklist. Of the 24 instruments, 16 were rated strong (nine of the 15 adult instruments and seven of the 10 pediatric instruments). Overall, insufficient information was reported in the included studies to assess the content validity, construct validity, responsiveness, and test-retest reliability of most instruments identified in both adult and child populations. Quality assessment results for the adult instruments are presented in Table 1 and for the pediatric instruments in Table 2.

**Table 1:** Adult PRO instruments

			Psychometric Properties					
Instrument	Aim	Items and Scoring	Developed for SCD Patients	Content Validity (SCD-specific)	Internal Reliability (Cronbach’s α)	Test-retest Reliability	Construct Validity	Responsiveness
Coping, Self-efficacy, Self-esteem								
Multidimensional Health Locus of Control Scale (MHLC) [18]	To assess respondents’ beliefs about whether their health status is determined by their own actions, the actions of other individuals, or is determined by fate or chance	18 items across 3 subscales (internal, chance, and external) rated on a 6-point Likert scale; scoring system not reported	No	Unclear	Strong (0.82)	Unclear	Unclear	Unclear
Rosenberg’s Self-esteem Scale (SES) [18]	To measure global self-esteem	10 items scored on a 4-point Likert scale; higher scores correspond to higher self-esteem	No	Unclear	Strong (0.85)	Unclear	Unclear	Unclear
Sense of Mastery Scale (SOM) [18]	To assess general sense of life control and mastery	7-item instrument on a 4-point Likert scale; scoring system not reported	No	Unclear	Good (0.77)	Unclear	Unclear	Unclear
Sickle Cell Disease Self-efficacy Scale (SCD-SES) [18, 22]	To assess respondent’s ability to function on a day-to-day basis and to manage SCD symptomatology	9 items ranked on a 5-point Likert scale; higher scores indicate greater self-efficacy	Yes	Unclear	Strong (0.80–0.89)	Unclear	Unclear	Unclear
Sickle Cell Transition Intervention Programs Skills Checklists [25]	To assess the transition readiness of patients with SCD	5 knowledge skills sets (medical, educational/vocational, health benefits, social, independent) and 3 psychological checklists (self-efficacy, sickle cell stress, feelings about transition); scoring system not reported	Yes	Good	Good (0.46–0.86)	Unclear	Unclear	Unclear
Simple Rathus Assertiveness Scale-Short Form (SRAS-SF) [21]	To measure a patient’s assertiveness in the health care setting	19 items ranked on a 6-point Likert scale; higher scores indicate higher levels of assertiveness	No	Unclear	Strong (0.85)	Unclear	Unclear	Unclear
Pain								
PAINReportIt [23]	To help clinicians better understand the experience and etiology of pain in their patients	Patient marks areas of pain on a body outline drawing, circles words to describe pain quality and pattern, and writes narrative text to indicate activities that increase or reduce the pain, and selects pain severity indicators; scoring system not reported	No	Unclear	Unclear	Unclear	Unclear	Unclear
Sickle Cell Disease Pain Burden Interview-Youth^a^ (ages 7–21) (SCPBI-Y) [26]	To assess the impact of pain on physical, social/community, and emotional aspects of daily function	7 items ranked on a 5-point Likert scale; higher scores indicate higher pain burden	Yes	Good	Strong (0.89)	Good	Good	Unclear
West Haven-Yale Multidimensional Pain Inventory (WHYMPI) [17]	To assess patients’ ability to cope with chronic pain	52 items ranked on a 7-point Likert scale; scoring system not reported	No	Unclear	Good (0.64–0.86)	Unclear	Unclear	Unclear
Depression								
Beck Depression Inventory-Fast Screen (BDI-FS) [20]	To measure symptoms of depression	7 items ranked on a 4-point Likert scale, indicating frequency of symptom experienced over previous week; higher scores indicate more depressive symptoms	No	Unclear	Strong (0.81)	Unclear	Unclear	Unclear
Family Impact								
Parental Bonding Instrument (PBI) – Maternal Version [22]	To assess adults’ perceptions of maternal overprotection and care prior to the age of 16 years	25 items; score system not reported	No	Unclear	Strong (0.83–0.89)	Unclear	Weak	Unclear
Quality of Life								
Adult Sickle Cell Quality of Life Measurement System (ASCQ-Me) [24]	To assess HRQL in adult patients with SCD	4 item sets (cognitive, emotional, social functioning, and physical impact) and 5 additional items assessing pain episode severity and frequency; scoring system not reported	Yes	Strong	Strong (0.92–0.96)	Unclear	Good	Unclear
Spirituality								
Spiritual Well-being Scale [17]	To assess the general spirituality pertaining to existential and religious well-being	20 items scored on a 6-point Likert scale; scoring system not reported	No	Unclear	Strong (0.82–0.88)	Unclear	Unclear	Unclear
Stigma								
Sickle Cell Disease Health-related Stigma Scale (SCD-HRSS) [20]	To assess the stigma patients with SCD perceive from the general public, physicians, and family	30 items ranked on a 6-point Likert scale; higher scores indicate higher perceived stigma	No	Unclear	Strong (0.84)	Unclear	Unclear	Unclear
Treatment Satisfaction								
Adult Sickle Cell Quality of Life Measurement Quality of Care (ASCQ-Me QOC) [19]	To measure patients’ perceived quality of received health care services	27 items on four domains (access, provider communication, ED care, and ED pain treatment); scoring system not reported	Yes	Good	Good (0.70–0.86)	Unclear	Unclear	Unclear

^a^As this instrument includes young adults up to age 21 years, it was included in both the Adult and Pediatric categories

Note: “Weak” indicates poor performance (e.g., evidence of poor reliability) or a weakness that should be considered within the trial design (e.g., requires significant input by research team to administer, or no availability of language translations); “Good” indicates adequate or moderate performance (e.g., adequate reliability) or only mild limitations (e.g., availability of a small number of language translations, absence of evidence in a minority of adult patients (e.g., older adults)); “Strong” indicates excellent performance on all reported indicators (e.g., all subscales report excellent reliability; evidence) or notable advantages for use within a trial (e.g., freely accessible, wide range of language translations); “Unclear” indicates where no or insufficient evidence was reported to assess, or where evidence reported was conflicting (e.g., some subscales show excellent reliability while others did not)

Abbreviations: *HRQL* health-related quality of life, *SCD* sickle cell disease

**Table 2:** Pediatric PRO Instruments

			Psychometric Properties					
Instrument	Aim	Items and Scoring	Developed for SCD Patients	Content Validity (SCD-specific)	Internal Reliability (Cronbach’s α)	Test-retest Reliability	Construct Validity	Responsiveness
Health-related Quality of Life								
PedsQL*™* Generic Core Scales [28, 34, 36]	To assess HRQL of children as young as 2 years (proxy report) and children as young as 5 years (self-report)	23 items rated on a 5-point Likert scale; higher scores indicate more problems	No	Unclear	Strong (0.80–0.90 parent-report; 0.93 self-report)	Unclear	Unclear	Unclear
PedsQL*™* SCD Module [36]	To assess quality of life that is specific to assessing the health issues relevant to pediatric patients with SCD	43 items rated by a 5-point Likert scale; higher scores represent better quality of life	Yes	Unclear	Strong (0.95–0.97)	Unclear	Unclear	Unclear
PedsQL*™* Multidimensional Fatigue Scale [35, 36]	To measure fatigue experienced during the past 1 month across a variety of pediatric populations	18 items rated on a 5-point Likert scale; higher scores represent better quality of life	No	Unclear	Strong (0.95 parent-report; 0.90 self-report)	Unclear	Unclear	Unclear
Patient Reported Outcomes Measurement Information System (PROMIS) [29]	To measure a pediatric patient’s health attributes of depressive symptoms over the previous 7 days	156 items over 8 domains (physical functioning mobility, physical functioning upper extremity, pain interference, fatigue, depressive symptoms, anxiety, peer relationships, and anger) rated on a 5-point Likert scale; higher scores signify worse severity for depression, anxiety, anger, fatigue, and pain interference and better functioning for physical functioning mobility, physical functioning upper extremity, and peer relationships	No	Unclear	Unclear	Unclear	Good	Unclear
Pain								
Faces Pain Scale-Revised (FPS-R) [31]	To assess pain intensity in pediatric populations experiencing acute pain	6 faces depicting different levels of increasing pain intensity	No	Good	Unclear	Unclear	Unclear	Unclear
Sickle Cell Disease Pain Burden Interview-Youth (SCPBI-Y) [26]	To assess the pain burden among children and adolescents with SCD over the previous month	7 items ranked on a 5-point Likert scale; higher scores indicate higher pain burden	Yes	Good	Strong (0.89)	Good	Good	Unclear
Coping, Self-efficacy								
Coping Strategy Questionnaire (CSQ) [30]	To measure coping with pain in adults, adolescents, and young children	13 subscales, each with 6 items rated on a 7-point Likert-type scale; higher scores indicate greater pain	No	Unclear	Unclear	Unclear	Unclear	Unclear
Sickle Cell Self-efficacy Scale (SCSES) [27]	To assess adults and adolescent’s self-appraisals of their ability to engage in daily functional activities despite having SCD	9 items rated on a 5-point Likert scale; higher scores indicate greater self-efficacy	Yes	Unclear	Strong (0.87)	Unclear	Weak	Unclear
Functioning								
Youth Acute Pain Functional Ability Questionnaire (YAPFAQ) [37]	To assess physical function and functional recovery in youth undergoing acute and procedural pain	40 items rated on a 5-point Likert scale; higher scores indicate greater difficulty performing functional activities	No	Unclear	Strong (0.92)	Unclear	Unclear	Unclear
Family Impact								
Psychosocial Assessment Tool 2.0 (PAT2.0) [32]	To measure psychosocial risk in families of a child newly diagnosed with cancer	7 subscales; higher scores indicate many stressors	No	Unclear	Strong (0.84)	Unclear	Unclear	Unclear

Note: “Weak” indicates poor performance (e.g., evidence of poor reliability) or a weakness that should be considered within the trial design (e.g., requires significant input by research team to administer, or no availability of language translations); “Good” indicates adequate or moderate performance (e.g., adequate reliability) or only mild limitations (e.g., availability of a small number of language translations, absence of evidence in a minority of adult patients (e.g., older adults)); “Strong” indicates excellent performance on all reported indicators (e.g., all subscales report excellent reliability; evidence) or notable advantages for use within a trial (e.g., freely accessible, wide range of language translations); “Unclear” indicates where no or insufficient evidence was reported to assess, or where evidence reported was conflicting (e.g., some subscales show excellent reliability while others did not)

Abbreviations: *HRQL* health-related quality of life, *SCD* sickle cell diseas

### PRO instruments in adults with SCD

The SLR identified 10 publications [17–26] reporting on psychometric properties of 15 PRO instruments validated in adults with SCD in the US. Of these instruments, six assessed coping, self-efficacy, or self-esteem [18, 21, 22, 25], three assessed patient pain [17, 23, 26], and one each assessed depression [20], family impact [22], quality of life [24], spirituality [17], stigma [20], and treatment satisfaction [19]. Five instruments had been developed specifically for adults with SCD [18, 19, 22, 24–26]. Most of the included studies did not provide sufficient information on the psychometric properties to assess construct or content validity, test-retest reliability, or responsiveness of the instruments concerned. However, most studies reported strong or good internal reliability and consistency. None of the included studies provided information regarding the threshold of minimally important change (sometimes called minimal important difference [MID]) in health status for any of the instruments reviewed. An overview of the three psychometric properties included in this assessment – validity, reliability, and responsiveness – is given below. Additional details about the identified adult PRO instruments are provided in Table 1.

#### Validity

##### Content validity

Four of the 15 instruments, which measured self-efficacy [25], pain [26], quality of life [24], and treatment satisfaction [19], reported sufficient information to assess content validity. Of these, three instruments [19, 25, 26] were rated good, while one [24] was rated strong, indicating a higher ability of the instrument to adequately reflect the construct being measured. Three instruments were specifically developed for adults with SCD [19, 24, 25]; one additional instrument was developed including young adults up to age 21 years [26]. There was not adequate information on the remaining 11 instruments to assess content validity.

##### Construct validity

Two instruments (assessing pain [26] and quality of life [24]) had good construct validity, indicating a higher degree to which the scores of the health-related PRO instrument are consistent with the hypothesis. Both of these instruments were developed for patients with SCD. One instrument [22], assessing family impact and not developed specifically for SCD, had weak construct validity. For the remaining 12 instruments, there was insufficient information available to assess their construct validity.

#### Reliability

##### Internal reliability

Most instruments (14 out of 15) provided sufficient information to assess internal consistency reliability. Of the instruments developed specifically for patients with SCD, two instruments evaluating self-efficacy [25] and treatment satisfaction [19] had good internal consistency reliability (Cronbach’s alpha: 0.46–0.86; of note certain instruments reported reliability across domains, and so the instrument was rated good overall, but some domains may have higher Cronbach’s alpha scores). Three instruments (evaluating self-efficacy [18, 22], pain [26], and quality of life [24]) had strong internal consistency reliability (Cronbach’s alpha 0.80–0.96). Two instruments that were not developed for SCD had good internal consistency reliability (measuring self-esteem [18] and pain [17]; Cronbach’s alpha: 0.64–0.86), while seven instruments had strong internal consistency reliability (measuring coping and self-esteem [18, 21], depression [20], family impact [22], spirituality [17], and stigma [20]; Cronbach’s alpha: 0.81–0.89).

##### Test-retest reliability

Only one SCD-specific instrument [26] assessing pain had good test-retest reliability, indicating consistency in the test over time. For the remaining instruments, the available information was insufficient to assess their test-retest reliability.

#### Responsiveness

For none of the identified instruments was there sufficient information available to assess their responsiveness to change in the measured construct.

### PRO instruments in children with SCD

Ten PRO instruments that had been validated in children with SCD in the US were identified across 12 studies [26–37]. Four of these instruments were related to the assessment of generic HRQL [28, 29, 33–36]; two instruments each assessed children’s pain [26, 31] and coping mechanisms with SCD [27, 30]; and one instrument each assessed the functional impact of experiencing pain [37] and the family impact of caring for a child with SCD [32]. Overall, only three of these instruments were developed specifically for children with SCD [26, 27, 36]. Most of the included studies provided no information on the psychometric properties of the instruments they reported on, in terms of the construct and content validity, test-retest reliability, or responsiveness. Furthermore, the reviewed studies did not assess the threshold of MID in health status for any of the instruments. However, internal consistency reliability was considered to be good for most of the instruments reviewed. An overview of the three psychometric properties (validity, reliability, and responsiveness) is given below. Additional details about the identified pediatric PRO instruments can be found in Table 2.

#### Validity

##### Content validity

Two instruments assessing pain had good content validity, indicating that they were an adequate reflection of the construct to be measured. Of the two, one instrument was developed for children with SCD [26], while the other was not [31]. For the remaining eight instruments, there was insufficient information for assessment of content validity.

##### Construct validity

Two instruments, one measuring generic HRQL (not SCD-specific) [29] and one measuring pain (SCD-specific) [26] reported good construct validity. One instrument developed specifically for children with SCD to measure self-efficacy [27] had weak construct validity. Seven identified instruments did not provide adequate information to assess this component.

#### Reliability

##### Internal reliability

For seven instruments, there was sufficient information to assess internal consistency reliability. Three of these instruments were developed specifically for children with SCD and measured HRQL [36], pain [26] and self-efficacy [27]; all reported strong internal consistency reliability (Cronbach’s alpha: 0.87–0.97). Four instruments, measuring HRQL [28, 34–36], functioning [37], and family impact [32], were not specific to SCD. These instruments also have strong internal consistency reliability, with Cronbach’s alpha ranging from 0.80 to 0.95.

##### Test-retest reliability

Only one instrument [26] examining pain, was rated as having good test-retest reliability. This instrument was developed specifically for patients with SCD. For the other nine instruments, insufficient information was reported to evaluate this component.

#### Responsiveness

For none of the identified instruments was sufficient information available to assess their responsiveness to change.

## Discussion

SCD represents a major challenge for patients, their families, and health care professionals. As a lifelong debilitating condition punctuated by severe, potentially life-threatening acute crises, it poses multiple threats to health and well-being. Against this background, and to inform the conduct of future randomized controlled trials in patients with SCD, the current study aimed to provide insights into the psychometric properties of validated PRO instruments used to-date in the disease. Specifically, it systematically identified and evaluated relevant US-based studies that reported on and critiqued PROs spanning HRQL, key symptoms, and attitudinal responses to SCD in children with the condition, their caregivers, and adult patients.

Treatment cost and the impact of treatment on overall health care use and costs (i.e., budget impact) are primary considerations when making coverage and reimbursement decisions [38]. Traditionally, US payers have not considered PROs to inform decisions on health care in this setting [39]. However, the market access landscape is changing, and PROs may now be poised to play a more important role in payer decisions, as evidenced by the recognition that PROs are important for evaluating symptoms and therapy impact on functioning [40] and increased patient engagement and participation in treatment decision-making. Assessing the patient perspective, in terms of PROs, is considered one of the primary outcomes to focus on and incorporate into all clinical trials proposing novel interventions, devices, or pharmaceuticals that aim for FDA and other regulatory or reimbursement approval [15]. However, a significant challenge in PRO research is demonstrating the measurement value of these tools that best describes the patient’s experience and what is considered as “acceptable measurement criteria” by regulatory and reimbursement bodies [40, 41]. Use of poorly developed PRO measures with inadequate psychometric evidence or those designed for a purpose that differs from their actual use can have significant implications and lead to distorted, inaccurate, or equivocal findings [42, 43]. Instruments should therefore be chosen based on relevance and their applicability in the context of the proposed disease area to produce reliable estimates of patients’ experiences. Although the match between the content coverage and content validity is important, convincing evidence is also needed to confirm the reliability, validity, and responsiveness of PRO measures used in clinical trials. The FDA has displayed an interest in patient-centered drug development in patients with SCD, through the Patient-focused Drug Development initiative [10]. This program aims to gather patient perspectives on SCD, specifically the effects that most impact patients, current available therapies, and participation in clinical trials.

It is also important to note the increase in health technology assessment activities by groups that provide US payers with evaluations related to coverage and reimbursement. Such activities currently focus on clinical efficacy and economic outcomes or budget impact, with limited emphasis on PROs and HRQL. However, it is likely that, in the future, health technology assessment valuations to inform US payers may directly incorporate patient perspectives and efficacy as assessed through PROs. For that reason, this SLR aimed to include only PRO instruments either being developed for use or being validated in a SCD population. Based on the SLR of evidence in a US-based population, guidance on use of currently available PROs and recommendations for further research are listed below.

### Guidance for PRO use in SCD populations based on SLR findings

- Consider using the PedsQL™ SCD Module to assess SCD-specific impact in children. This instrument allows for evaluation of various concepts, including pain, fatigue, productivity, activity, and emotion.
- Consider using the Sickle Cell Disease Pain Burden Interview-Youth (SCPBI-Y) to assess the impact of painful crises in children aged over 7 years.
- Use a short generic pain assessment tool, such as the Brief Pain Inventory, or numerical rating scales for assessing pain intensity for adults.
- Use the Adult Sickle Cell Quality of Life Measurement System (ASCQ-Me) to assess patient-reported HRQL in adults. This instrument allows for evaluation of patients’ pain, fatigue, productivity, activity, and emotion.
- Use the ASCQ-Me Quality of Care (QOC) instrument to assess patient perceptions of accessibility of care and the quality of interactions with health care providers.

### Recommendations for further PRO research in SCD populations

- There should be validation of a generic preference-based PRO instrument to evaluate general aspects of HRQL.
- There should be validation of specific instruments for younger children (ages 5–12 years) (caregiver report version of the SCPBI-Y and Psychosocial Assessment Tool 2.0) and for adults (ASCQ-Me, SCIPBI-Y).
- For relevant outcomes of interest (function, psychological well-being), there should be validation of the PedsQL™ 4.0 Generic Core Scales for children and the ASCQ-Me for adults.
- There should be validation (or, if none exist, development) of instruments that assess other outcomes of interest (e.g., cognitive impairment, school/work performance and attendance, treatment satisfaction).
- There should be piloting of administration of identified instruments using electronic devices (e.g., tablets, phone apps).

## Conclusion

There appears to be only limited evidence available on the psychometric properties of PRO instruments developed for use in patients with SCD. It is also crucial to note is that among the instruments reviewed, none was found to sufficiently capture all the impacts of SCD and its complications on patients’ HRQL for use as key trial endpoints. Further research is therefore required to develop and validate PRO instruments for assessing the impact of SCD on adults and children, and their caregivers. The proposed recommendations and the other key information and insights from this SLR could help to inform future clinical trials for patients with SCD in the US.

## Additional files

Additional file 1: Database Search Strategy. Search strategies used in electronic literature databases. (DOCX 16 kb)

Additional file 2: PICOS-T Inclusion and Exclusion Criteria. Inclusion and exclusion criteria applied to the identified studies. (DOCX 17 kb)

## Abbreviations

ASCQ-Me: Adult Sickle Cell Quality of Life Measurement System
COSMIN: COnsensus-based Standards for the selection of health Measurement INstruments
ED: Emergency department
FDA: Food and Drug Administration
HRQL: Health-related quality of life
MID: Minimal important difference
PedsQL: Pediatric Quality of Life Inventory
PRISMA: Preferred Reporting Items for Systematic Reviews and Meta-Analyses
PRO: Patient-reported outcome
QOC: Quality of Care
RBC: Red blood cell
SCD: Sickle cell disease
SCPBI-Y: Sickle Cell Disease Pain Burden Interview-Youth
SLR: Systematic literature review
US: United States
VOC: Vaso-occlusive crises
