# Supplementary material for: Systematic literature review and assessment of patient-reported outcome instruments in sickle cell disease
Source: Health Qual Life Outcomes. 2018 May 21;16:99. doi: 10.1186/s12955-018-0930-y (PMC5963009; doi:10.1186/s12955-018-0930-y)
Supplement: Supplementary file 1 — Database Search Strategy. Search strategies used in electronic literature databases. (DOCX 16 kb) [file 12955_2018_930_MOESM1_ESM.docx]

Additional File 1

Table 1. Database Search Strategy

| **Number** | **Query** | **Results (March 23, 2017)** |
| --- | --- | --- |
| 1 | drepanocytosis:ab,ti OR 'sickle cell anemia'/exp OR 'sickle cell anemia':ab,ti OR 'sickle cell anaemia':ab,ti OR 'sickle cell disease':ab,ti OR 'sickle cell hemoglobinopath*':ab,ti OR 'sickle cell crisis':ab,ti | 34,023 |
| 2 | 'cohort analysis'/exp OR 'cross-sectional study'/exp OR reglst*:ab,ti OR 'database':ab,ti OR 'cohort study':ab,ti OR 'cohort studies':ab,ti OR 'cohort analysis':ab,ti OR 'cohort analyses':ab,ti OR 'observational':ab,ti OR 'longitudinal':ab,ti OR 'retrospective':ab,ti OR 'prospective':ab,ti OR 'cross sectional':ab,ti OR 'real world':ab,ti OR psychometr*:ab,ti OR validat*:ab,ti | 2,614,213 |
| 3 | 'randomized controlled trial'/exp OR 'randomization'/exp OR random*:ab,ti OR 'rct':ab,ti OR 'controlled trial':ab,ti OR 'clinical trial':ab,ti OR 'single blind procedure'/exp OR 'double blind procedure'/exp OR 'crossover procedure'/exp OR 'cross over':ab,ti OR 'crossover':ab,ti OR 'placebo'/exp OR 'placebo':ab,ti OR (doubl* AND blind*:ab,ti) OR (singl* AND blind*:ab,ti) OR ('open':ab,ti AND label*:ab,ti) | 1,636,783 |
| 4 | 'quality of life'/exp OR 'quality of life':ab,ti OR 'quality-of-life':ab,ti OR hrql:ab,ti OR 'patient reported outcome':ab,ti OR 'patient-reported outcome':ab,ti OR 'patient reported outcomes':ab,ti OR 'patient-reported outcomes':ab,ti OR 'utility':ab,ti OR 'functional status':ab,ti OR 'physical function':ab,ti OR 'caregiver burden':ab,ti OR 'eq-5d':ab,ti OR 'eq 5d':ab,ti OR euroqol:ab,ti OR 'health utility scale':ab,ti OR 'health utility index':ab,ti OR hui:ab,ti OR 'standard gamble':ab,ti OR 'time trade off':ab,ti OR 'time-trade-off':ab,ti OR tto:ab,ti OR rosser*:ab,ti OR 15d:ab,ti OR '15 d':ab,ti OR '15 dimension':ab,ti OR 12d:ab,ti OR '12 d':ab,ti OR '12 dimension':ab,ti OR 5d:ab,ti OR '5 d':ab,ti OR '5 dimension':ab,ti OR 6d:ab,ti OR '6 d':ab,ti OR '6 dimension':ab,ti OR (short:ab,ti AND form:ab,ti OR shortform:ab,ti OR sf:ab,ti AND (thirtysix:ab,ti OR thirty:ab,ti AND six:ab,ti OR 36:ab,ti)) OR ('health measurement':ab,ti AND scale:ab,ti) OR ('health measurement':ab,ti AND questionnaire:ab,ti) OR 'health status'/exp OR 'health survey'/exp OR 'index of wellbeing':ab,ti OR 'quality of wellbeing':ab,ti OR qwb:ab,ti OR 'rating scale':ab,ti OR 'medical outcomes study':ab,ti OR 'visual analogue scale':ab,ti OR vas:ab,ti OR 'patient rated response to therapy':ab,ti OR 'sickle cell disease health related stigma scale':ab,ti OR 'sickle cell disease quality of life' OR 'sickle cell disease quality of life questionnaire' OR 'scdqol' OR 'pain coping questionnaire' OR pcq OR 'pediatric quality of life inventory' OR pedsql OR questionnaire OR scale OR inventory OR instrument OR well-being OR ‘well being’ OR wellbeing | 2,229,966 |
| 5 | [english]/lim | 25,942,116 |
| 6 | [editorial]/lim OR [erratum]/lim OR [letter]/lim OR [note]/lim OR [short survey]/lim OR [review]/lim OR 'in vitro study'/de OR 'clinical protocol'/de OR 'case report'/exp OR 'case report':ti OR 'conference abstract'/it | 10,480,615 |
| 7 | [animals]/lim NOT [humans]/lim | 5,111,802 |
| 8 | (#1 AND (#2 OR #3) AND #4 AND #5) NOT #6 NOT #7 | 524 |
| 9 | (#1 AND (#2 OR #3) AND #4 AND #5) NOT #6 NOT #7 AND [1997-2017]/py | 498 |
| 10 | [article in press]/lim | 185,183 |
| 11 | #1 AND (#2 OR #3) AND #4 AND #5 NOT #6 NOT #7 AND #10 | 6 |
| 12 | #9 OR #11 | 504 |
