# Supplementary material for: Systematic literature review and assessment of patient-reported outcome instruments in sickle cell disease
Source: Health Qual Life Outcomes. 2018 May 21;16:99. doi: 10.1186/s12955-018-0930-y (PMC5963009; doi:10.1186/s12955-018-0930-y)
Supplement: Supplementary file 2 — PICOS-T Inclusion and Exclusion Criteria. Inclusion and exclusion criteria applied to the identified studies. (DOCX 17 kb) [file 12955_2018_930_MOESM2_ESM.docx]

Additional File 2

PICOS-T Inclusion and Exclusion Criteria

| **Category** | **Inclusion Criteria** | **Exclusion Criteria** |
| --- | --- | --- |
| **Population** | - Children (aged 5–17 years) - Caregivers of children with SCD - Adults with SCD (≥18 years) | Publications reporting on patient populations in the following categories:   - Children <5 years with SCD - Patients without a diagnosis of SCD - Children and adults with SCD (humanistic burden evidence) |
| **Interventions/ comparators** | Treatments for the management of SCD and its symptoms (including watchful waiting) | Studies evaluating treatments for comorbid conditions |
| **Outcomes*** | **Psychometric properties of PRO instruments as they relate to current FDA guidelines**   - Reliability - Internal consistency reliability - Test-retest reliability - Validity - Content validity - Construct validity - Reproducibility - Responsiveness - Interpretability - Applicability - Burden of completion - Respondent - Investigator | Publications that do not report data on outcomes of interest |
| **Study designs** | RCTs, non-randomized interventional studies, observational studies, psychometric development or evaluation studies | Publications with other study designs |
| **Temporal** | Studies published from January 1997 until March 23, 2017 | Studies published before January 1997 |
| **Geographical** | Studies reporting data for populations in the US   - Studies with ≥80% population in the US were considered eligible. | Studies that do not report data separately for populations in the US or studies where <80% of the study sample is a US population |
| **Publication types** | Full-text journal articles | Publications of the following types:   - Narrative publications - Systematic/non-systematic literature reviews - Case studies - Case reports - Editorials - Conference abstracts |
| **Language** | Studies published in English | Publications not in English |

* Studies were only excluded for not reporting relevant outcomes at the full-text screening phase.

Abbreviations: FDA = Food and Drug Administration; PICOS-T = Population, Intervention/Comparator, Outcome, Study design, Time point; PRO = patient-reported outcome; RCT = randomized controlled trial; SCD = sickle cell disease; US = United States
